# Occlusive Myocardial Infarction: A Practical Review of STEMI-Negative Coronary Occlusion in Acute Coronary Syndromes

**DOI:** 10.3390/medicina62101843

**Published:** 2026-09-23

**Authors:** Gil Mashiah, Reem Elbassel, Lior Zornitzki

**Affiliations:** 1 Department of Cardiology, Tel Aviv Sourasky Medical Center, Tel Aviv 6423906, Israel; reemelbassel@gmail.com; 2 Division of Cardiology, Schulich Heart Centre, Sunnybrook Health Sciences Centre, Toronto, ON M4N 3M5, Canada; lior.zoz@gmail.com; 3 Gray Faculty of Medical and Health Sciences, Tel Aviv University, Tel Aviv 6997801, Israel

**Keywords:** acute coronary syndrome, occlusion myocardial infarction, STEMI equivalent, electrocardiography, coronary occlusion, reperfusion, artificial intelligence, acute myocardial infarction

## Abstract

ST-segment elevation myocardial infarction (STEMI) and non-ST-segment elevation myocardial infarction (NSTEMI) remain the dominant operational categories for acute coronary syndrome triage because they are simple, widely implemented, and closely linked to reperfusion pathways. However, diagnostic ST-segment elevation and acute coronary occlusion are not synonymous. A clinically important subgroup of patients classified as NSTEMI have total or near-total culprit-artery occlusion and may experience delayed angiography despite ongoing myocardial ischemia. This structured narrative review examines the rationale, evidence, electrocardiographic patterns, quantitative bedside criteria, and emerging technologies relevant to occlusion myocardial infarction (OMI), with particular emphasis on STEMI-negative coronary occlusion. Conventional STEMI criteria are highly specific but incompletely sensitive for acute coronary occlusion, particularly in posterior or circumflex infarction, early anterior occlusion, left bundle branch block, ventricular paced rhythm, and transient reperfusion. Recognition of OMI therefore requires integration of ECG morphology, reciprocal changes, serial ECG evolution, clinical presentation, troponin kinetics, and adjunctive imaging rather than reliance on ST-segment thresholds alone. Practical patterns include hyperacute T waves, the de Winter pattern, posterior OMI, modified Sgarbossa-positive conduction abnormalities, subtle anterior OMI, and selected high-risk ischemic patterns. Artificial intelligence may improve scalable detection of subtle occlusion patterns, although external validation, workflow integration, explainability, and prospective outcome studies remain necessary. Current evidence supports OMI as a promising complementary diagnostic framework rather than an established replacement for guideline-based STEMI/NSTEMI classification. Prospective implementation studies are required to determine whether OMI-guided pathways improve clinical outcomes without unacceptable false-positive catheterization-laboratory activation.

## 1. Introduction

Acute coronary syndromes (ACSs) require rapid assessment, and the initial 12-lead electrocardiogram (ECG) often determines whether a patient undergoes immediate reperfusion. Current guidelines continue to use the STEMI/NSTE-ACS classification as a simple and widely implemented framework for emergency triage [1,2,3].

However, acute coronary occlusion does not always produce ST-segment elevation that meets conventional STEMI criteria. ECG findings depend on the location and extent of ischemia, timing of presentation, collateral flow, spontaneous reperfusion, and underlying conduction or repolarization abnormalities [4,5]. Consequently, some patients with an acutely occluded culprit artery may be classified as NSTEMI and undergo delayed reperfusion.

The concept of occlusion myocardial infarction (OMI) aims to identify these patients. This review focuses on the limitations of conventional STEMI criteria, ECG patterns associated with STEMI-negative coronary occlusion, and the evidence needed to determine whether earlier recognition and treatment of OMI improves clinical outcomes.

## 2. Materials and Methods

This structured narrative review focused on acute coronary occlusion in patients who do not meet conventional STEMI criteria. PubMed/MEDLINE was searched from database inception through 4 July 2026 using combinations of OMI/NOMI, acute coronary occlusion, occluded NSTEMI, relevant ECG patterns, and AI-assisted ECG interpretation with Boolean operators (AND/OR). Google Scholar was searched through the same date as a supplementary citation-discovery source. Because this search was iterative, the exact number of screened results was not prospectively recorded. International ACS guidelines, consensus documents, and reference lists of relevant studies were also reviewed.

English-language peer-reviewed studies addressing angiographically confirmed coronary occlusion, diagnostic performance of ECG criteria, outcomes of occluded NSTEMI, invasive-treatment timing, or AI-assisted detection were considered. Primary angiographic and diagnostic-accuracy studies, independent validation cohorts, systematic reviews, meta-analyses, randomized trials, and seminal ECG reports were prioritized. Non-peer-reviewed publications and studies without direct relevance to the diagnosis or management of acute coronary occlusion were excluded. Quantitative findings were preferentially derived from primary studies or systematic reviews.

The initial search and relevance filtering were performed by G.M., and the final selection and interpretation were reviewed by all authors. After deduplication and initial relevance filtering, 68 publications remained. Following title/abstract review, 59 publications underwent full-text assessment, and 50 were included in the final narrative synthesis (Figure 1). Because OMI definitions and study designs varied substantially, the evidence was synthesized narratively. Key diagnostic studies were interpreted with particular attention to patient selection, reference standards, disease prevalence, external validation, and potential spectrum bias, informed by relevant QUADAS-2 domains.

## 3. From STEMI/NSTEMI to an Occlusion-Focused Approach

The STEMI/NSTEMI classification is effective for rapidly identifying patients who require immediate reperfusion. However, the absence of ST-segment elevation does not exclude acute coronary occlusion. Angiographic studies have shown that a substantial proportion of patients classified as NSTEMI have an occluded culprit artery, with reported rates varying according to the study population and angiographic definition [6,7,8,9,10]. These patients tend to have larger infarcts and worse outcomes than NSTEMI patients without occlusion [6,7].

OMI describes acute myocardial infarction caused by a persistent, intermittent, or recently reperfused culprit-artery occlusion that may still be reperfusion-sensitive. Definitions vary between studies, most commonly according to pre-intervention TIMI flow and evidence of recent occlusion [11,12]. No internationally accepted definition of OMI currently exists.

A key limitation of the STEMI/NSTEMI approach is that acute coronary occlusion may not produce classic STEMI criteria on the initial ECG. Instead, the ECG may show hyperacute T waves, subtle ST-segment changes, reciprocal abnormalities, or reperfusion patterns before or without diagnostic ST elevation. These findings may be particularly important when the clinical presentation and serial ECG changes suggest ongoing ischemia.

## 4. Evidence Base and Critical Appraisal

### 4.1. Angiographic Prevalence, Patient Characteristics, Biomarkers, and Prognosis

Angiographic NSTEMI studies provide the strongest evidence that acute coronary occlusion can be missed by STEMI criteria. Meta-analyses have shown that occluded NSTEMI is associated with larger infarcts, lower LVEF, more cardiogenic shock, and worse short- and long-term outcomes [6,7]. Circumflex involvement is also more common, which may partly explain why some occlusions are not recognized on the initial 12-lead ECG [7]. The available data are mainly observational and are influenced by differences in patient selection, timing of angiography, and the definition of occlusion.

Occluded NSTEMI has also been associated with persistent symptoms, ST-segment depression, larger biomarker release, and greater hemodynamic compromise [9,10]. However, none of these features reliably identifies vessel occlusion in an individual patient. Troponin reflects myocardial injury rather than coronary patency: an early value may still be low during acute occlusion, while a high or rising value may indicate a larger infarct without distinguishing persistent occlusion from spontaneous reperfusion [7].

Independent cohorts report similar findings, although occlusion rates vary according to the angiographic definition used [10,12]. Taken together, these data support an integrated assessment based on ECG findings, symptoms, biomarkers, and adjunctive imaging rather than any single clinical or biochemical marker.

### 4.2. Diagnostic Accuracy, Spectrum Bias, and Predictive Values

A 2024 meta-analysis of three diagnostic-accuracy studies involving 23,704 patients found that ST-segment elevation had a pooled sensitivity of 43.6% and specificity of 96.5% for acute coronary occlusion. An OMI/NOMI interpretation strategy increased sensitivity to 78.1%, with a specificity of 94.4% [13]. However, the evidence was limited by the small number of studies and substantial heterogeneity.

In the Meyers study, expert OMI interpretation increased sensitivity from 41% with STEMI criteria to 86%, with a small reduction in specificity from 94% to 91% [11]. The study was retrospective and case-control, and one-third of the study population had OMI, reflecting an enriched cohort compared with an unselected emergency population. A related retrospective study found that only 60% of adjudicated OMI met STEMI criteria and that STEMI-negative OMI was less likely to undergo catheterization within 90 min [14]. The DIFOCCULT study similarly identified non-STEMI patients with occlusion-associated ECG findings and greater myocardial injury [15].

The prevalence of OMI is considerably lower in unselected chest-pain populations. In a consecutive cohort of 24,511 emergency-department patients with chest pain, OMI was identified in only 1.9% of patients [16]. This contrasts markedly with the 33% prevalence in the Meyers cohort, which was enriched with OMI cases. As a result, predictive values and the apparent diagnostic yield of OMI interpretation in that study cannot be directly extrapolated to routine emergency-department populations.

Overall, these studies suggest that an occlusion-focused approach may improve recognition of acute coronary occlusion, but they do not establish that OMI-guided catheterization improves clinical outcomes.

### 4.3. Evidence from Invasive Angiography-Timing Trials

Randomized trials in NSTE-ACS have examined whether earlier angiography, rather than a delayed or standard invasive strategy, improves clinical outcomes. TIMACS compared angiography within 24 h with a delayed strategy after 36 h, while VERDICT compared angiography within 12 h with a standard strategy at 48–72 h [17,18]. Neither trial showed a significant overall reduction in its primary clinical outcome, although higher-risk patients may have derived greater benefit.

RIDDLE-NSTEMI evaluated immediate angiography within 2 h versus a delayed strategy at 2–72 h in patients with NSTEMI, while EARLY compared angiography within 2 h with a 12–72 h strategy in patients with NSTE-ACS [19,20]. The observed benefits were mainly reductions in myocardial infarction or recurrent ischemia, without a clear mortality benefit. An individual-patient meta-analysis similarly found no overall mortality reduction with an early invasive strategy [21].

Importantly, these trials were designed to examine the timing of angiography in NSTE-ACS, not to identify patients with acute coronary occlusion. They therefore do not establish whether immediate reperfusion is beneficial specifically in STEMI-negative OMI. Whether prospectively identifying this subgroup and treating it earlier improves outcomes therefore remains unanswered.

## 5. Practical ECG Recognition of STEMI-Negative OMI

ECG remains the earliest practical tool for recognizing acute coronary occlusion, but interpretation should extend beyond conventional ST-elevation thresholds or isolated named patterns. The distribution and proportionality of ST-T changes, reciprocal abnormalities, QRS morphology, dynamic ECG changes, and comparison with previous tracings may all contribute to identifying STEMI-negative OMI. Representative ECG examples, adapted from published cases [22,23,24,25,26,27], are shown in Figure 2, and the main quantitative criteria and diagnostic limitations are summarized in Table 1.

**Posterior OMI.** Posterior OMI may present as horizontal ST depression, particularly when maximal in V1–V4, with posterior leads V7–V9 providing additional diagnostic information [28,29].

**De Winter pattern.** The de Winter pattern consists of upsloping precordial ST depression with tall, symmetric T waves and is strongly associated with LAD occlusion [30,31].

**Hyperacute T waves.** Hyperacute T waves may precede overt ST elevation and should be interpreted in relation to QRS morphology and the clinical presentation [32].

**LBBB and modified Sgarbossa criteria.** In LBBB, proportional ST-segment changes are particularly important. The modified Sgarbossa criteria improve recognition of acute occlusion by incorporating excessive discordance in addition to the original concordant criteria [33,34,35].

**Subtle anterior OMI.** Quantitative formulas may help distinguish subtle anterior LAD occlusion from benign ST elevation, although they should be used as adjuncts to clinical and ECG interpretation rather than in isolation [36,37].

**Wellens pattern.** Wellens morphology is characterized by biphasic or deeply symmetric T-wave inversion, typically in V2–V3 and sometimes extending across the anterior precordial leads. It usually reflects critical LAD disease or reperfusion rather than persistent occlusion [38].

**Aslanger pattern and South African flag sign.** The Aslanger pattern consists of isolated ST elevation in lead III, ST depression in V4–V6 with positive or terminally positive T waves, and greater ST elevation in V1 than V2. The South African flag sign shows ST elevation in I, aVL, and V2 with reciprocal ST depression in lead III. Both may identify important ischemia outside classic contiguous-lead STEMI criteria but remain less extensively validated [39,40].

**Diffuse ST depression with ST elevation in aVR.** This pattern indicates widespread subendocardial ischemia but is not specific for acute left-main occlusion [1,41].

These findings should therefore be interpreted together with symptoms, serial ECGs, biomarkers, and other clinical findings.

**Figure 2:**
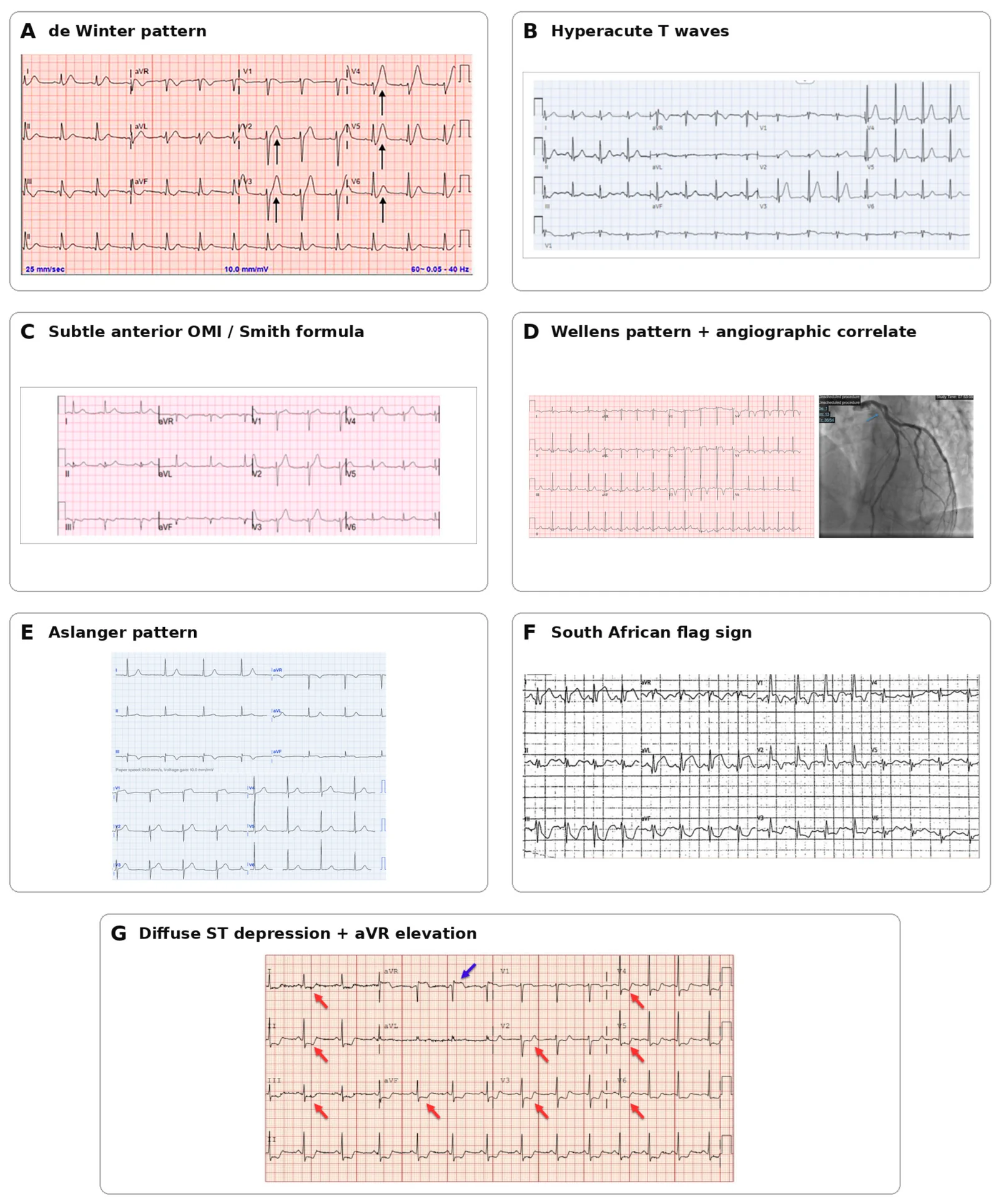
Representative ECG patterns associated with STEMI-negative OMI: (**A**) de Winter pattern, with black arrows indicating the characteristic upsloping precordial ST-segment depression and tall, symmetric T waves, adapted from [22]; (**B**) hyperacute T waves, adapted from [23]; (**C**) subtle anterior OMI, adapted from [23]; (**D**) Wellens pattern, with the blue arrow indicating the culprit LAD lesion on angiography, adapted from [24]; (**E**) Aslanger pattern, adapted from [25]; (**F**) South African flag sign, adapted from [26]; and (**G**) diffuse ST-segment depression with ST-segment elevation in aVR, with red arrows indicating diffuse ST-segment depression and the blue arrow indicating ST-segment elevation in aVR, adapted from [27]. All panels were reproduced or adapted in accordance with the applicable licenses of the original publications.

**Table 1:** Quantitative and operational ECG criteria for STEMI-negative OMI and related high-risk ischemic patterns.

Pattern	Operational/Quantitative Criteria	Clinical Interpretation/Pitfall
**Posterior OMI** [28,29]	Horizontal ST depression maximal in V1–V4; posterior confirmation with ST elevation ≥ 0.5 mm in V7–V9 in the appropriate setting.	Often LCx/RCA posterior territory. Record posterior leads when suspicion is high; do not dismiss maximal anterior ST depression as nonspecific.
**de Winter** [30,31]	Upsloping J-point ST depression, commonly ~1–3 mm in V1–V6, merging into tall symmetric T waves; aVR elevation may occur; no universal single cutoff.	Usually LAD occlusion. Pattern may evolve into/out of classic STEMI; morphology and clinical context matter.
**Hyperacute T waves** [32]	Broad, symmetric, bulky T waves disproportionate to QRS/baseline. A software-derived morphology score mean ≥ 0.7 in ≥2 consecutive leads was highly specific but insensitive.	Early occlusion; compare with prior ECG/QRS amplitude. Hyperkalemia and benign variants are important mimics; use serial ECGs.
**Modified Sgarbossa** [33,34,35]	Concordant STE ≥ 1 mm; concordant STD ≥ 1 mm in V1–V3; or discordant STE ≥ 1 mm with ST/S ratio ≤ −0.25.	Acute occlusion may be masked by LBBB. Apply proportional criteria; do not call these ECGs uninterpretable.
**Subtle anterior LAD occlusion** [36,37]	3-var: 1.196 × STE60V3 + 0.059 × QTc-B − 0.326 × RAV4; ≥23.4 favors LAD occlusion. 4-var: 0.052 × QTc-B − 0.151 × QRSV2 − 0.268 × RAV4 + 1.062 × STE60V3; ≥18.2 favors LAD occlusion.	Use only in the intended benign-variant vs. subtle LAD-occlusion differential after excluding obvious ischemic features; external validation supports but does not make it a stand-alone catheterization-laboratory trigger.
**Terminal QRS distortion** [42]	J point ≥ 50% of R-wave amplitude in a qR complex or absence of S wave in an Rs complex in ≥2 adjacent anterior leads.	Associated with severe anterior ischemia/larger infarct; supportive sign, not a stand-alone OMI definition.
**Wellens** [38]	Pain-free/resolving-pain ECG with biphasic or deep symmetric anterior T-wave inversion, classically V2–V3, with little/no ST elevation.	Transient LAD occlusion/reperfusion or critical LAD lesion. Avoid stress testing; high risk persists after pain resolves.
**Aslanger** [39]	STE in III but not II/aVF; STD V4–V6 with positive/terminally positive T waves; STE V1 > V2.	High-risk inferior infarction pattern with concomitant diffuse ischemia; not a universally standardized guideline category.
**South African flag** [40]	STE in I, aVL and V2 with reciprocal inferior ST depression.	High-lateral/first-diagonal vector; useful teaching pattern but limited validation.
**Diffuse STD + aVR/V1 elevation** [1,41]	ESC high-risk pattern: STD ≥ 1 mm in ≥6 leads with STE in aVR and/or V1 suggests multivessel ischemia/left-main obstruction, especially with hemodynamic compromise.	Global subendocardial ischemia; not specific for acute left-main occlusion. Consider LM/proximal LAD/3-vessel disease and severe supply-demand mismatch.

## 6. Integrated Bedside Assessment: ECG, Clinical Features, Troponin, and Imaging

Recognition of STEMI-negative OMI should not rely on a single ECG or a single variable. The central clinical question is whether the overall presentation suggests an acutely occluded or dynamically occluding culprit artery that may still benefit from urgent reperfusion. Figure 3 summarizes a practical integrated approach. It is intended as a clinical framework rather than a prospectively validated decision rule.

Clinical features are most helpful when they suggest ongoing instability. Persistent or recurrent ischemic chest pain, diaphoresis, malignant ventricular arrhythmias, acute heart failure, shock, or dynamic ECG changes should all increase concern for ongoing occlusion and lower the threshold for urgent escalation under current ACS pathways [1,2]. By contrast, demographic associations reported in observational cohorts are not sufficiently specific to guide decisions in individual patients.

Serial ECG assessment is particularly important because coronary thrombosis and reperfusion are dynamic. Worsening ST-T abnormalities during symptoms support ongoing ischemia, whereas rapid normalization or evolution to reperfusion T-wave changes may indicate transient occlusion or spontaneous reopening. Comparison with prior tracings is especially useful in patients with LBBB, ventricular pacing, LVH, or chronic repolarization abnormalities. Additional leads should not be overlooked: right-sided leads are useful in inferior MI, particularly when right ventricular involvement is suspected, and posterior leads should be obtained when posterior infarction is suspected [1,3].

High-sensitivity cardiac troponin is essential for confirming myocardial injury and estimating infarct burden. Both hs-cTnI and hs-cTnT are widely used, and neither has been shown to be superior for identifying OMI specifically. Differences between assays are mainly related to analytical characteristics and assay-specific thresholds rather than their ability to determine culprit-artery patency. Higher values, and particularly peak concentrations, are generally associated with larger infarcts, but the peak is usually reached too late to guide the initial decision for urgent reperfusion. In early presenters, even a low initial troponin cannot exclude acute coronary occlusion. Serial measurements can strengthen or weaken the suspicion of a substantial evolving infarction but should be interpreted together with symptoms, ECG evolution, and clinical status [7].

Focused echocardiography may strengthen diagnostic confidence when the ECG is nondiagnostic by demonstrating a new regional wall-motion abnormality or by suggesting an alternative diagnosis. Because regional dysfunction develops rapidly during acute ischemia, a technically adequate study showing normal regional wall motion during ongoing symptoms makes a large ongoing ischemic territory less likely. It does not, however, completely exclude acute coronary occlusion, particularly with smaller territories, intermittent or recently reperfused occlusion, or limited image quality.

Echocardiography should support decision-making but should not delay angiography when the clinical and ECG findings already suggest a high likelihood of reperfusion-sensitive occlusion.

Prehospital recognition may be particularly important in STEMI-negative OMI, where expert ECG interpretation is often not immediately available. Paramedic education, repeat ECGs, and AI-assisted interpretation may help identify high-risk occlusion patterns earlier and improve triage.

## 7. Artificial Intelligence and Scalable OMI Recognition

AI may be useful in OMI because the ECG changes of acute coronary occlusion can be subtle and are not always captured by conventional STEMI criteria. In this setting, AI may serve as an additional ECG reader, particularly when expert interpretation is not immediately available.

Different approaches have been explored. Some models rely on predefined ECG measurements, while deep-learning models analyze the 12-lead waveform directly. Herman et al. developed a deep-learning model using 18,616 ECGs from 10,543 patients with suspected ACS and subsequently tested it in an international cohort. The model analyzed a standard 12-lead ECG to predict the presence of OMI and showed substantially higher sensitivity for detecting OMI than conventional STEMI criteria [43]. Other models have been studied in prehospital, emergency-department, catheterization-laboratory, and high-risk NSTE-ACS populations, with similarly encouraging results [16,44,45,46,47]. However, performance has varied between studies, partly because of differences in patient selection and in the definition of OMI.

Whether these results can be reproduced in routine clinical practice remains uncertain. Independent studies have reported promising findings [48], but a 2026 systematic review found that most AI-ECG studies in acute MI were retrospective and that external validation was performed in only a minority of studies [49]. Differences in patient populations, ECG equipment, and disease prevalence may all influence performance when a model is applied outside its original setting.

At present, AI is best viewed as a tool to support ECG interpretation rather than replace it. Most deep-learning models provide a probability of OMI without clearly showing which ECG features led to the prediction. Questions also remain regarding the appropriate alert threshold, interpretation of serial ECGs, false-positive catheterization-laboratory activation, and integration into routine workflow.

Finally, the available studies mainly show improved diagnostic performance rather than improved clinical outcomes. Prospective studies are needed to determine whether AI-assisted OMI recognition reduces missed occlusion or shortens time to reperfusion without substantially increasing unnecessary catheterization. Until such evidence is available, AI should remain a decision-support tool rather than an independent trigger for reperfusion.

## 8. Implementation, Limitations, and Research Priorities

Several challenges remain before an occlusion-focused approach can be incorporated into routine ACS pathways. Greater sensitivity may come at the cost of false-positive catheterization-laboratory activation, and interpretation of subtle ECG patterns may vary between clinicians. OMI should therefore complement, rather than replace, current STEMI/NSTE-ACS pathways.

A major limitation is the lack of a standardized OMI definition. For future research, we propose defining OMI primarily by an acute culprit lesion with pre-intervention TIMI 0–2 flow. TIMI 2 is included because it may reflect severe residual flow impairment or partial reperfusion after recent occlusion. Transient or reperfused OMI may also be considered when TIMI 3 flow is present together with objective evidence of recent occlusion or reperfusion. This remains an authors’ proposed research framework rather than a consensus definition.

The key unanswered question is whether earlier recognition of STEMI-negative OMI improves clinical outcomes. Prospective studies should assess time to reperfusion, infarct size, heart failure, mortality, missed occlusion, false-positive activation, and resource use. DIFOCCULT-3 represents an important prospective step in this direction [50].

## 9. Conclusions

The STEMI/NSTEMI framework remains essential for ACS triage but does not identify every acute coronary occlusion. Current evidence supports the existence of a clinically important STEMI-negative occlusive phenotype and suggests that an occlusion-focused ECG approach may improve recognition. However, the available evidence is largely observational, definitions remain heterogeneous, and it has not yet been shown that OMI-guided early intervention improves clinical outcomes.

OMI should therefore be viewed as a complementary framework rather than a replacement for current STEMI/NSTEMI classification. Clinical assessment should integrate ECG findings and serial changes with symptoms, hemodynamics, troponin, and imaging. AI may further support recognition, but prospective studies using standardized definitions and patient-centered outcomes are required before broader implementation can be recommended.

## Figures and Tables

**Figure 1:**
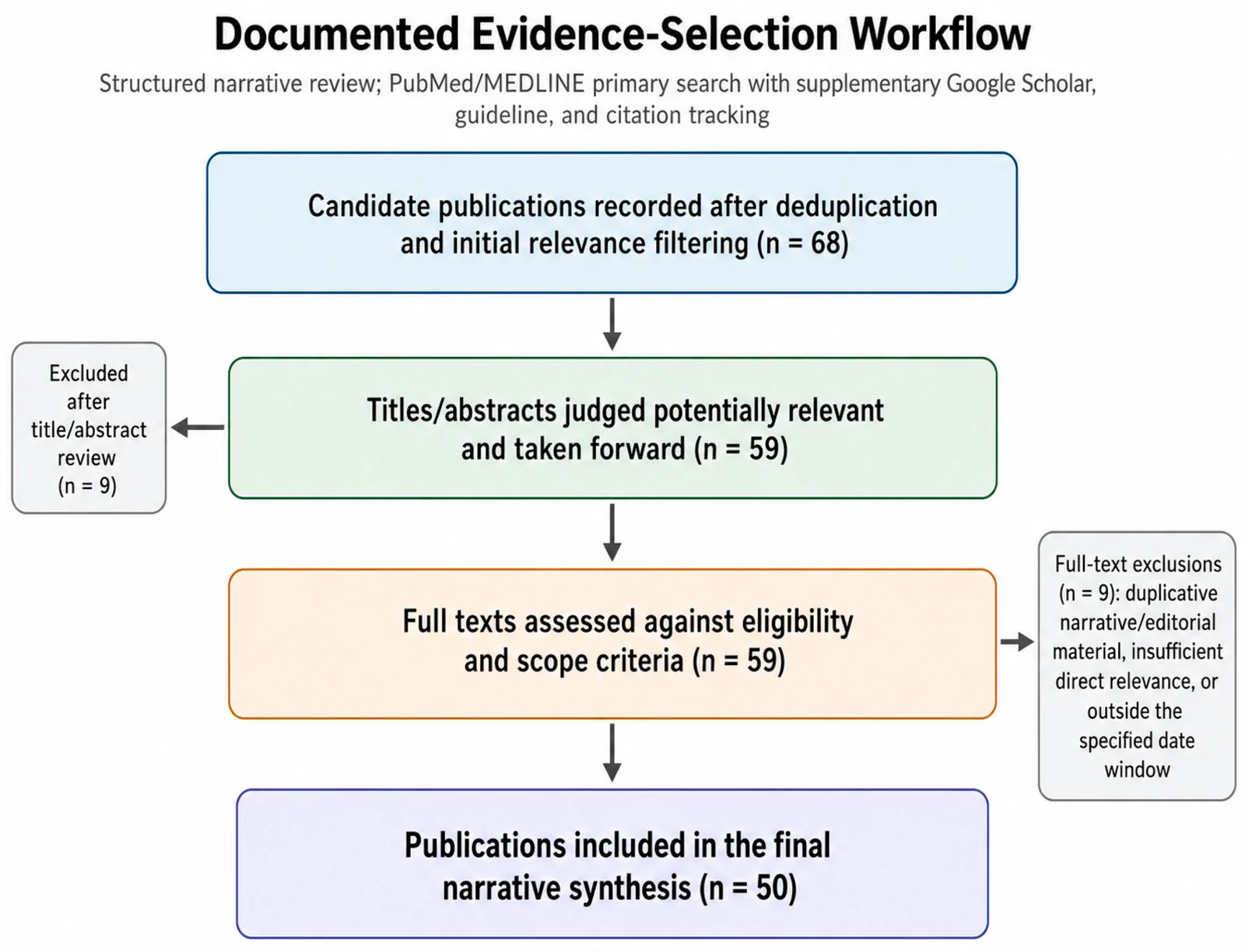
Study-selection flow diagram for the structured narrative review. The diagram documents article selection for a narrative synthesis and should not be interpreted as indicating that the review was conducted as a formal systematic review.

**Figure 3:**
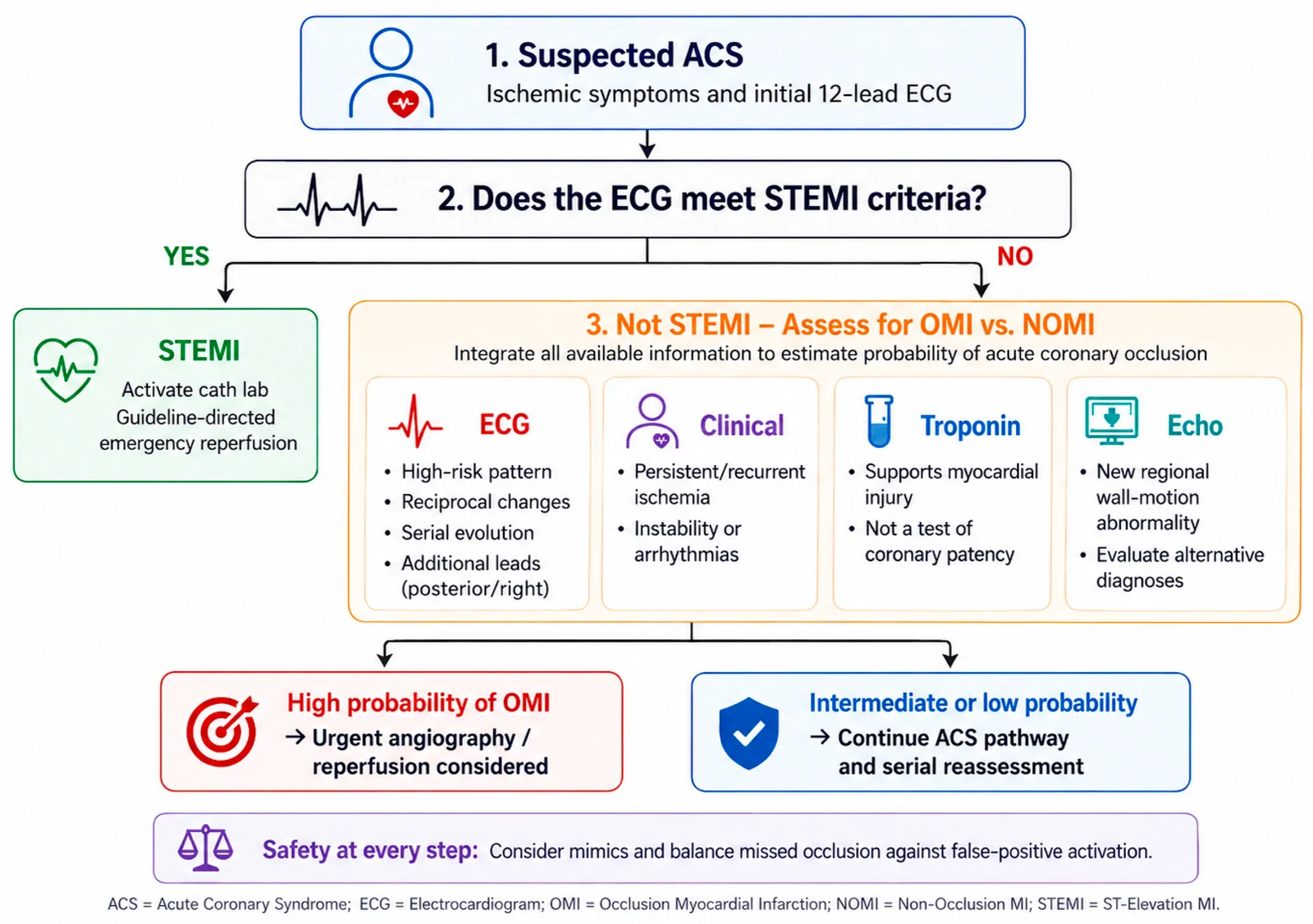
Integrated bedside framework for suspected STEMI-negative OMI. In patients with suspected ACS who do not meet conventional STEMI criteria, assessment should integrate ECG findings, clinical presentation, serial ECG changes, troponin kinetics, and focused echocardiography to estimate the likelihood of ongoing or recurrent coronary occlusion. The figure represents an authors’ proposed clinical framework and has not been prospectively validated as a decision rule.

## Data Availability

No new data were created or analyzed in this study. Data sharing is not applicable to this article.
